# Crystal-seeds induced construction of ZnO–ZnFe_2_O_4_ micro-cubic composites as excellent anode materials for lithium ion battery†

**DOI:** 10.1039/c8ra01785a

**Published:** 2018-05-01

**Authors:** Pei Pan, Ting Wang, Lihui Chen, Feng Wang, Xiong Yang, Caiqin Qin, Yu Ding

**Affiliations:** College of Chemistry and Materials Science, Hubei Engineering University Xiaogan 432000 China hbeukj@126.com dy9802@126.com; Hubei Collaborative Innovation Center for Advanced Organic Chemical Materials, Ministry-of-Education Key Laboratory for Synthesis and Applications of Organic Functional Molecules, Hubei University Wuhan 430062 China; School of Materials and Energy, Guangdong University of Technology Guangzhou 510006 China

## Abstract

This work aims at designing a fine assembly of two different transition metal oxides with a distinct band-gap energy into a bi-component-active hetero-structure to enhance the hetero-interface interactions and synergetic functionalities of bi-components to improve electrochemical performance. Herein, a facile marriage of crystal-seeds induction and hydrothermal reactions has been utilized to fabricate ZnO–ZnFe_2_O_4_ micro-cubic composites. Benefiting from the synergetic effects of the bi-functional components and their unique hetero-junction structure, the ZnO–ZnFe_2_O_4_ micro-cubic composites exhibit a significant improvement in lithium storage performance. The reversible capacity is retained at a value of 811 mA h g^−1^ after 200 cycles at a current density of 100 mA g^−1^. Even at high current densities of 1 and 5 A g^−1^, the electrodes are still able to deliver capacities of 584 and 430 mA h g^−1^ after 200 cycles, respectively.

## Abstract 2

This work aims at designing a fine assembly of two different transition metal oxides with a distinct band-gap energy into a bi-component-active hetero-structure to improve electrochemical performance.

## Graphical abstract

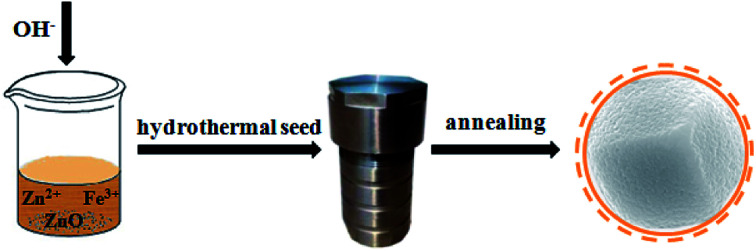

## Introduction

Rechargeable lithium ion batteries (LIBs) with high energy density and superior power density are regarded as one of the most promising types of energy device for application in electric vehicles and portable electronics.^1–3^ Nevertheless, the carbon material based anodes of commercial LIBs still suffer from safety issues and relatively low lithium storage capacity. Among the various alternative materials, transition metal oxides have mostly been preferred for use as anode materials in LIBs, because of their abundant availability, relatively higher theoretical capacity, and low cost.^4,5^ Ternary ferrite oxide systems, owing to the synergetic effects of their complex chemical compositions and natural structure, have exhibited significantly improved electrochemical performance as electrode materials for LIBs.^6–9^ Among them, ZnFe_2_O_4_, as a more competitive electrode material for LIBs, has received tremendous attention owing to its low toxicity, high natural abundance, low lithiation voltage (∼1.5 V), and high theoretical capacity (∼1072 mA h g^−1^).^10–12^ In particular, the combination of an alloying/dealloying mechanism and conversion reactions during the lithiation/delithiation process of ZnFe_2_O_4_ can contribute to the extra capacity and improve its electrochemical performance. To date, various microstructure characteristics and morphologies of ZnFe_2_O_4_ have been successfully investigated for advanced LIBs, such as nanospheres,^11^ nanowires,^13^ nanoparticles,^14^ sub-microcubes,^15^ and so on.^16,17^ However, as an electrode material for LIBs, ZnFe_2_O_4_ still suffers from poor electronic conductivity and inferior cycling stability.

Currently, the fine assembly of two kinds of transition metal oxides with distinct band-gap energies to be converted into a bi-component-active hetero-structure could improve our understanding of the inherent properties of hetero-interface interactions and synergetic functionalities of bi-components to improve electrochemical performance.^10^ Furthermore, the bi-component-active hetero-structure can enhance the surface reaction kinetics and facilitate charge transport to improve the lithium storage performance of electrode materials because of its internal electric field at hetero-interfaces. In previous reports, it has been confirmed that unique hybrid architectures, such as SnS_2_@SnO_2_,^18^ Fe_2_O_3_@SnO_2_,^19^ ZnO@Co_3_O_4_,^20^ CuO@SnO_2_,^21^ and ZnO–ZnFe_2_O_4_,^22^ could achieve high specific capacity, remarkable rate performance, and excellent cycling stability compared to any single component.

Among the different transition metal oxide materials, ZnO, a multifunctional n-type semiconductor with a wide band gap (3.37 eV), has been widely applied in electronic devices because of its high electrical conductivity and excellent chemical–thermal stability. ZnO can offer effective pathways for electron transport and provide a strong mechanical support. An integrated hetero-structure electrode material, constructed with different metal oxides with different band-gap energies, can successfully enhance the internal electric field at the hetero-interfaces, resulting in an improvement in the surface reaction kinetics and facilitating charge transport to improve the electrochemical activity of the electrode materials.^23^ Based on the inspiration mentioned above, an integrated n-type ZnO and ternary ferrite oxide p-type ZnFe_2_O_4_ (with a narrow band gap of 1.9 eV) hetero-structure could result in an excellent electrode material for designing LIBs. In particular, the existence of alloying/dealloying (Li–Zn) processes and conversion reactions (Zn–Li_2_O–ZnO/Fe–Li_2_O–Fe_2_O_3_) will enhance the electrochemical performances.^10^ However, the two challenges that are vital to overcome to enhance the ultimate electrochemical performance of the hetero-structured electrode are attainment of a homogeneous dispersion of bi-functional component for a hetero-interface, and controlling and optimizing the morphology to make the electrode's properties appealing for LIBs.

In the present work, we have developed a facile marriage of crystal-seeds induced synthesis and hydrothermal reactions to fabricate ZnO–ZnFe_2_O_4_ micro-cubic composites. This fabrication strategy results in well-dispersed hetero-structured bi-functional components, and a fine assembly of the morphology of the composites *via* micro-cubic ZnO seeds. Benefiting from the unique structure and bi-functional components, the ZnO–ZnFe_2_O_4_ micro-cubic composites exhibit excellent electrochemical performance as anode materials for LIBs.

## Experimental section

### Synthesis of cubic ZnO crystal-seeds

A certain amount of Zn(NO_3_)_2_·6H_2_O was dissolved in 100 mL of saturated oxalate solution, along with simultaneous stirring, leading to the formation of a white precipitate. The as-obtained precipitate was filtered with deionized water and dried in a vacuum oven at 60 °C for 24 h, then finally calcined at 800 °C for 6 h at a heating rate of 5 °C min^−1^ in a muffle furnace to obtain the ZnO crystal-seeds.

### Synthesis of micro-cubic ZnO–ZnFe_2_O_4_ composites

ZnO–ZnFe_2_O_4_ was synthesized through a facile hydrothermal route. ZnO crystal-seeds were dispersed in 25 mL of deionized water by ultrasonication for 30 min, then 12 mmol of FeCl_3_·6H_2_O and 6 mmol of ZnCl_2_ were added and stirred for 30 min. Subsequently, 1 M NaOH solution was slowly dripped into the mixture under continuous stirring to adjust the pH to 10–11. Next, the suspension was transferred into a 100 mL Teflon-lined stainless-steel autoclave and maintained at 160 °C for 12 h. The generated precursor was collected by filtration with deionized water and ethanol and dried in a vacuum oven at 60 °C for 24 h. Finally, the obtained sample was calcinated at 700 °C for 4 h at a heating rate of 2 °C min^−1^, to obtain the final product. For the sake of comparison, a series of syntheses were performed by changing the mass ratios of ZnO seed for the total weight of FeCl_3_·6H_2_O and ZnCl_2_ to 0%, 1.25%, 2.5% and 5%, and the resulting products were denoted ZnFe-1, ZnFe-2, ZnFe-3 and ZnFe-4, respectively.

## Material characterizations

The structures and morphologies of the as-prepared samples were characterized by X-ray powder diffraction (XRD, Bruker, D8 diffractometer), scanning electron microscopy (SEM, Hitachi S3400N) and transmission electron microscopy (TEM, FEI Tecnai G20). The element types and valence states were analyzed by the X-ray photoelectron spectroscopy (XPS, Kratos Analytical-A Shimadzu group company).

### Electrochemical measurements

Electrochemical measurements were performed using coin-type CR2016 cells in a glove box filled with high purity argon. The electrode was prepared by mixing 80 wt% sample, 10 wt% polyvinylidene fluoride and 10 wt% Super-P-Li in *N*-methyl pyrrolidone. Next, the mixture was coated on the copper foil and dried in a vacuum oven at 100 °C for 12 h. Following this, the electrode was cut into 10 mm diameter discs which were used as the anode. Lithium foil was used as the counter electrode and Celgard 2400 porous polypropylene as the separator. The electrolyte comprised a solution of 1 M LiPF_6_ in a mixture of diethyl carbonate (DEC) and ethylene carbonate (EC) (1 : 1, v/v). The electrochemical performances of the cells were tested on LANHE CT2001A instruments and CHI 660E electrochemical workstations.

## Results and discussion

**Fig. 1:**
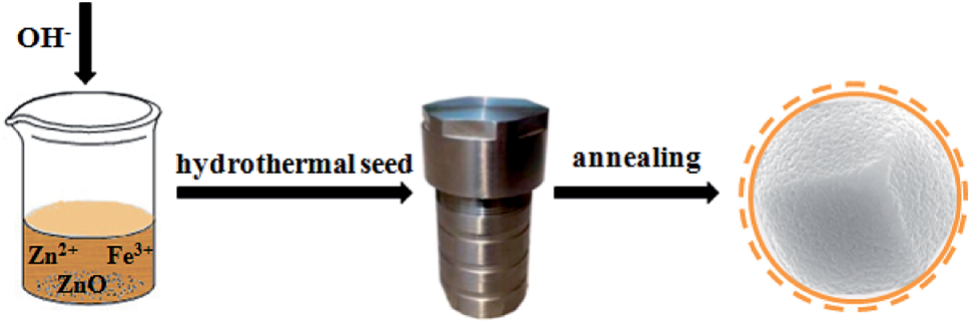
Schematic representation of the preparation of ZnO–ZnFe_2_O_4_ composites.

As shown in Fig. 1, the fabrication of ZnO–ZnFe_2_O_4_ micro-cubic composites comprises two steps: a crystal-seeds induced hydrothermal route followed by a thermal treatment. The cubic ZnO seeds were added into the reaction system, which could reduce the critical ability for the formation of a new phase and provide a large number of crystal nuclei to facilitate crystal growth. During the crystal nucleation, growth up-regulation and Ostwald ripening process, the expected micro-cubic ZnO–ZnFe_2_O_4_ crystals will be nucleated and eventually grow into cubic structures under the induction of ZnO seeds.^24^ To enhance the crystallinity of the samples, an annealing process was conducted, resulting in the formation of ZnO–ZnFe_2_O_4_ composites.

**Fig. 2:**
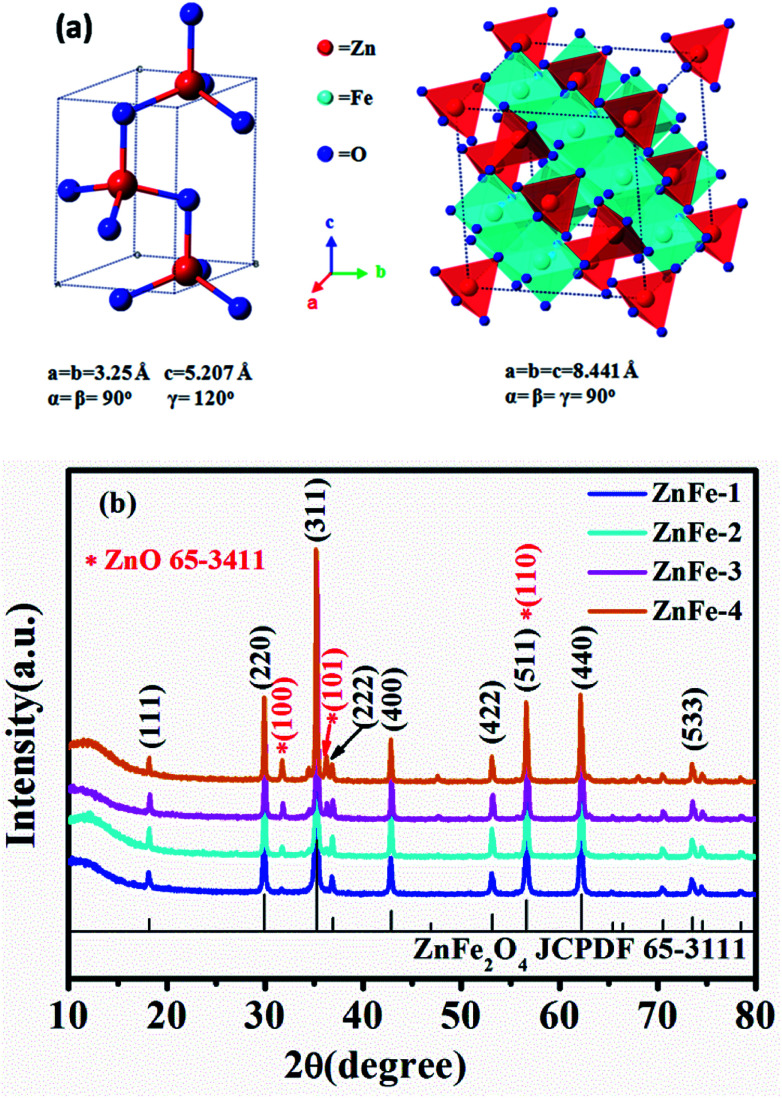
(a) Crystal structure of ZnO and ZnFe_2_O_4_. (b) XRD patterns of synthesized ZnO and ZnO–ZnFe_2_O_4_.

Fig. 2a depicts the crystal structures of ZnO and ZnFe_2_O_4_. The characteristic reflections exhibit an obvious two-phase crystallographic blend of hexagonal structured ZnO with a space group of *P*6_3_*mc* (186) and ZnFe_2_O_4_ with a cubic structure of *Fd*3*m* (227). The Zn and Fe atoms occupy the center of the tetrahedrons and the octahedrons are constituted by oxygen atoms. This results in a spinel type structure. Fig. 2b shows the XRD pattern of ZnO–ZnFe_2_O_4_ micro-cubic composites. The peaks at 18.2°, 29.9°, 35.3°, 36.9°, 42.9°, 53.2°, 56.7°, 62.2°, and 73.6° correspond to the planes (111), (220), (311), (222), (400), (422), (511), (440), and (533) of the polycrystalline ZnFe_2_O_4_ phase (JCPDS card no. 65-3111), respectively. The remaining peaks at 31.8°, 36.3°, and 56.6° can be indexed with the (100), (101) and (110) planes of ZnO (JCPDS card no. 65-3411). Fig. S1† indicates that the XRD pattern of the ZnO seed can be well indexed to the diffraction from (100), (002), (101), (102), (110), (103), (112) and (201) planes of the ZnO phase (JCPDS card no. 65-3411).

**Fig. 3:**
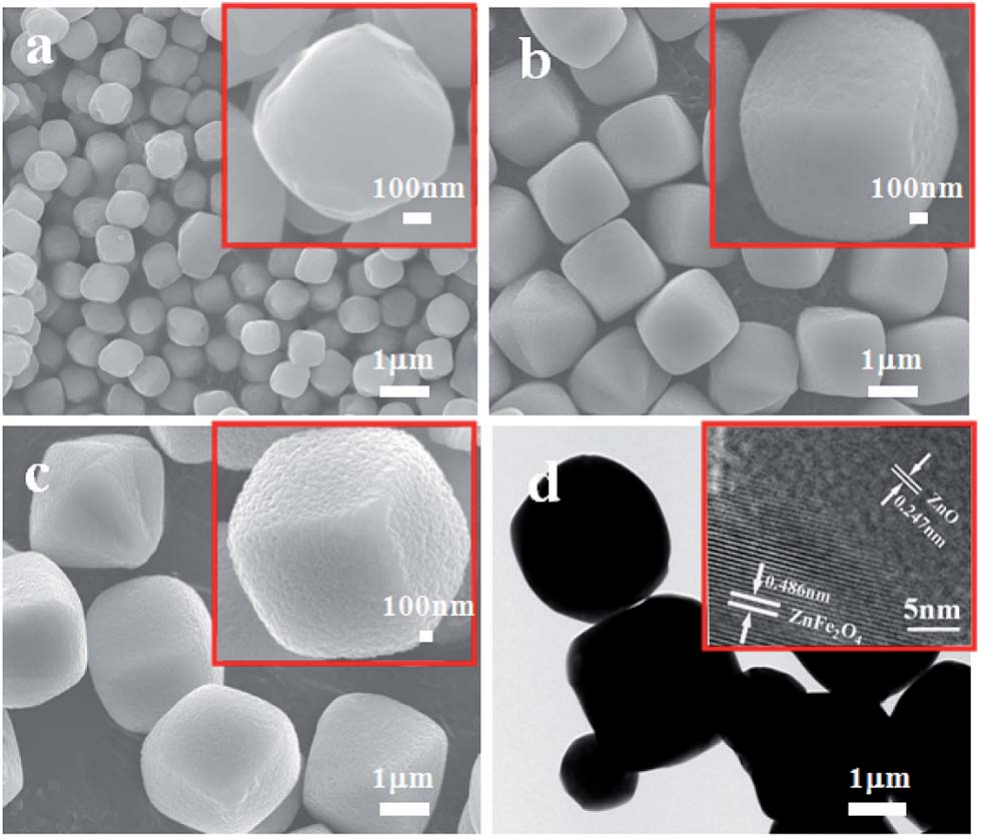
SEM images of ZnFe-2 (a), ZnFe-3 (b), and ZnFe-4 (c). TEM and HRTEM images of ZnFe-3 (d).

The morphology of the as-obtained ZnO–ZnFe_2_O_4_ composites was investigated by SEM and TEM. Fig. 3a–c show that the sample is composed of a number of micro-cubic particles of approximately 1.5 μm in size. The relatively broad size distribution of the micro-cubic particles could be attributed to the overlapping of the nucleation and growth processes at high temperatures with a long time period.^25^ The 0.247 and 0.486 nm lattice fringes in the ZnFe-3 could correspond to the (101) plane of ZnO and the (111) plane of ZnFe_2_O_4_, respectively.

**Fig. 4:**
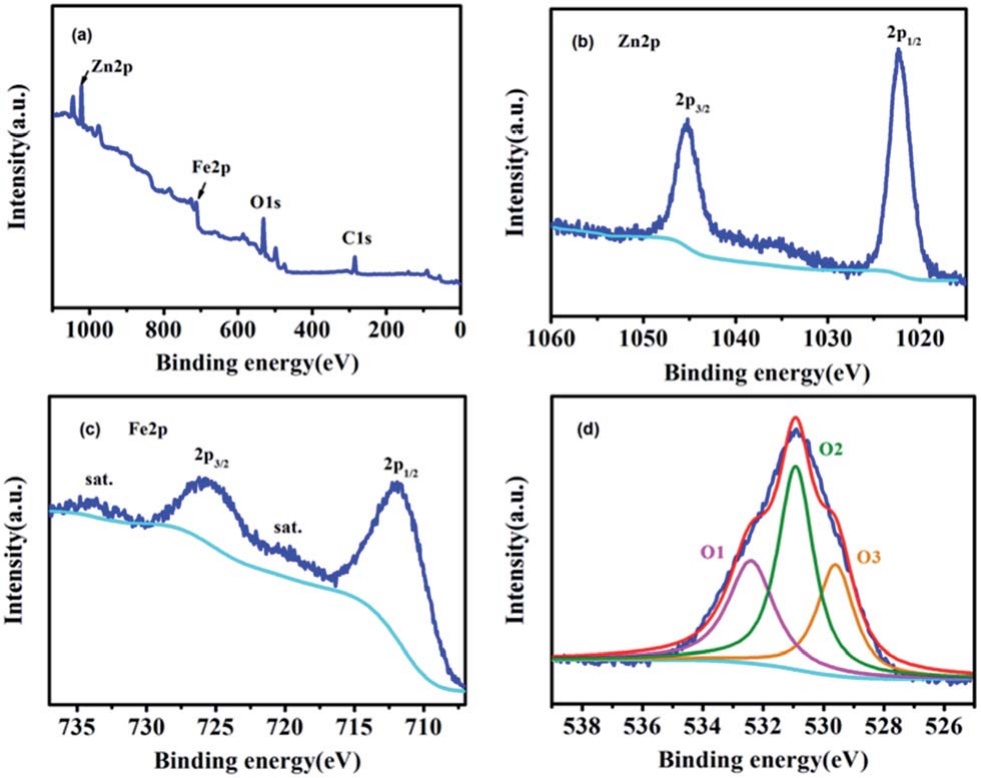
XPS spectra (a) survey spectrum, (b) Zn 2p, (c) Fe 2p, and (d) O 1s for ZnFe-3.

The XPS survey spectrum of ZnFe-3 and the high-resolution spectra of Zn, Fe, and O are shown in Fig. 4. The overview survey confirms the presence of Fe, Zn, and O elements (Fig. 4a). The Zn 2p spectrum displays two peaks at 1045.2 eV and 1022.3 eV with a binding energy of 22.9 eV originating from Zn 2p_3/2_ and Zn 2p_1/2_ (Fig. 4b).^26^ The two main peaks at 725.6 and 711.8 eV can be assigned to Fe 2p_1/2_ and Fe 2p_3/2_, and the other two peaks at 734 and 720.4 eV show good agreement with the shakeup satellites.^25,27^ The high-resolution spectrum of O 1s can be fitted into three peaks. To be specific, the peak at 529.6 eV (O1) can be ascribed to the typical lattice oxygen in the metal (Zn/Fe)–oxygen framework.^28^ The peak at 530.9 eV suggests the presence of other oxygen composites (O2), such as OH and H_2_O,^29^ and the peak at 532.4 eV (O3) can be attributed to a large number of defect sites having low oxygen co-ordination in the ZnO and ZnFe_2_O_4_ species.^29^

**Fig. 5:**
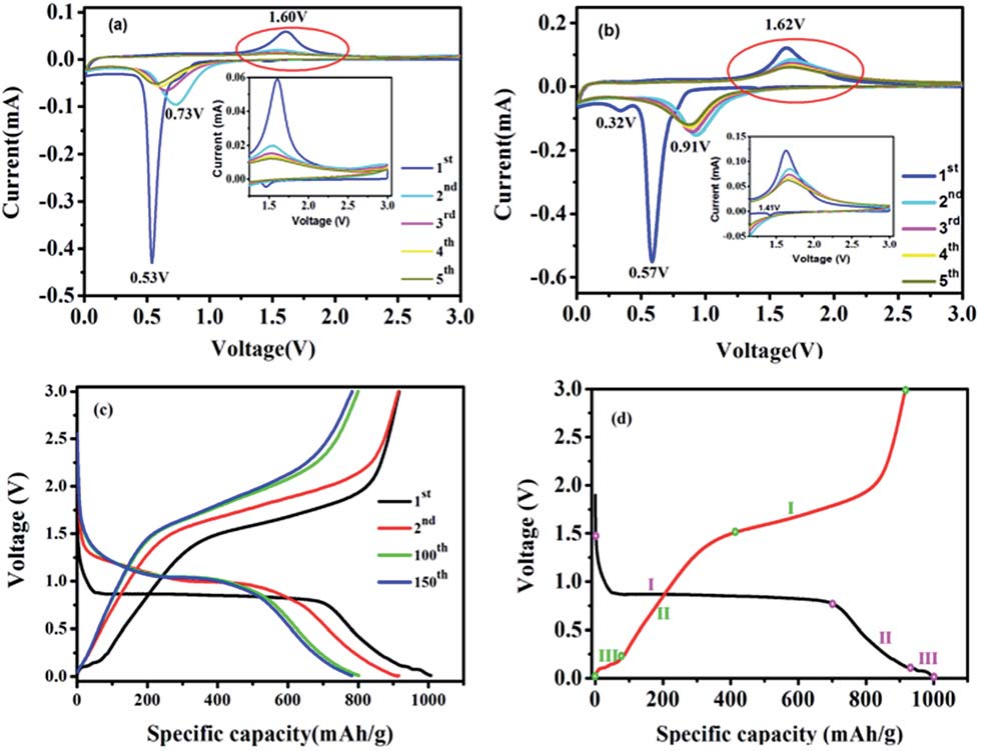
CV curves of ZnFe-1 (a) and ZnFe-3 (b) electrodes. The voltage-specific capacity profiles of ZnFe-3 (c) and the CV of the first cycle for ZnFe-3 (d) at 100 mA g^−1^.

The electrochemical performances of the ZnO–ZnFe_2_O_4_ micro-cubic composites were first evaluated by CV, which presents a better understanding of the redox reactions and the internal structural transformations occurring during the lithiation–delithiation cycles. The CV curves of micro-cubic ZnFe-3 and ZnFe-1 are shown in Fig. 5. During the first delithiation process, three broad reduction peaks were observed at 1.41, 0.57 and 0.32 V for ZnFe-3 (Fig. 5b). The anodic peak at 1.41 V is attributed to the formation of Li_*x*_ZnFe_2_O_4_. The two prominent peaks at 0.57 and 0.32 V can be associated with the reduction of Zn^2+^ and Fe^3+^ to metallic Zn^0^ and Fe^0^, along with the irreversible decomposition of the electrolyte to form the solid-electrolyte interface (SEI). In subsequent cycles, one main anodic peak shifts to ∼0.91 V, which is due to the structural rearrangement from the pulverization of the composites and the capacity loss which occurs during the process of charging. When the voltage decreases to about 0.05 V, deintercalation of Li^+^ from the Li–Zn alloy is evident.^30^

The broad anodic peak at 0.11 V belongs to the dealloying reaction of Li–Zn, and the one corresponding to ∼1.62 V can be attributed to the reaction of the metallic Zn and Fe into Zn^2+^ and Fe^3+^, respectively. During the subsequent cycles, the intensity and integral area of the peak decrease slightly with an increase in the cycling numbers for ZnFe-3, which obviously decreases for bare ZnFe-1 (Fig. 5a). When the voltage corresponding to the range from 1.0 to 3.0 V is enlarged, the intensity of the anodic peaks of ZnFe-1 is found to be almost negligible. These results indicate that the ZnO–ZnFe_2_O_4_ hybrid exhibits better electrochemical reversibility than the pristine ZnFe_2_O_4_ samples.

**Fig. 6:**
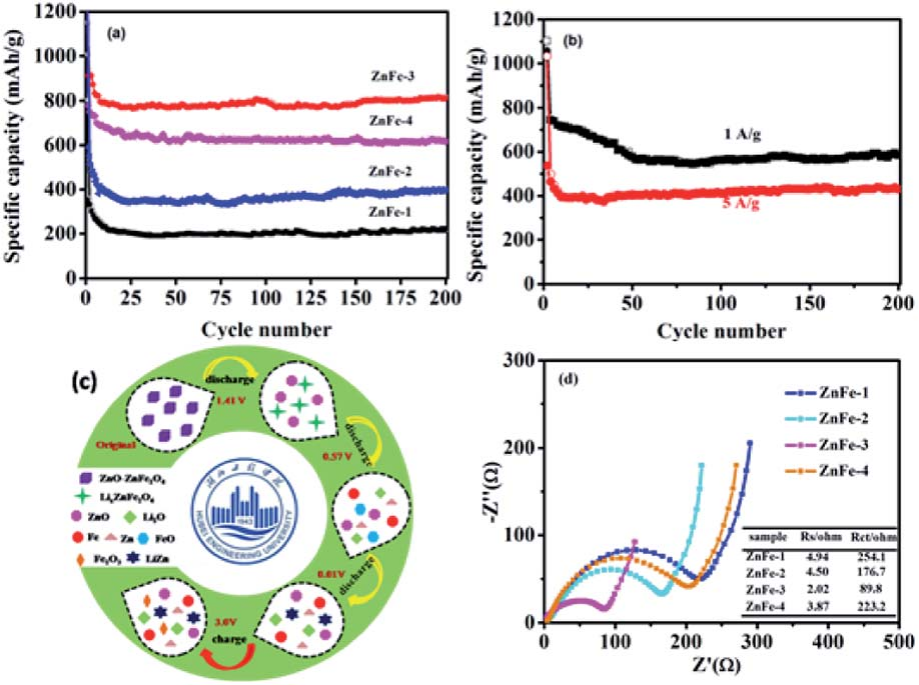
(a) The lithiation/delithiation performance of the ZnO–ZnFe_2_O_4_ composites at 100 mA g^−1^. (b) The lithiation and delithiation performance of ZnFe-3 at current densities of 1 and 5 A g^−1^. (c) The possible electrochemical mechanism of the ZnO–ZnFe_2_O_4_ composite electrode during the 1st discharge and charge cycle. (d) The EIS of ZnO–ZnFe_2_O_4_ composites.

Fig. 6a displays the lithiation/delithiation performance of the ZnO–ZnFe_2_O_4_ composite electrode at a current density of 100 mA g^−1^ in the range 0.01–3.0 V (*vs.* Li^+^/Li). The ZnFe-3 exhibits the best electrochemical performance with a high capacity retention and cycling stability. It delivers a superior capacity of 1007/910 mA h g^−1^ in the first cycle and retains a reversible capacity of 817/811 mA h g^−1^ even after 200 cycles. The electrochemical performance of the ZnO–ZnFe_2_O_4_ composites exhibits a far superior cycling stability compared with the ZnFe_2_O_4_ (ZnFe-1). The voltage-specific capacity profiles of ZnFe-3 are shown in Fig. 5c. The irreversible capacity during the initial cycle can be attributed to the formation of an SEI film and an incomplete conversion reaction. The coulombic efficiency of ZnFe-3 is 90.4%, which is a significant improvement over our early ZnFe_2_O_4_ systems.^31^ The first charge/discharge profile of ZnFe-3 is shown in Fig. 5d. Both the lithiation and delithiation curves are divided into three parts. The parts labeled I and II in the lithiation/delithiation processes can be assigned to the reaction equations:

1 ZnFe_2_O_4_ + *x*Li^+^ + *x*e^−^ → Li_*x*_ZnFe_2_O_4_,

and

2 Li_*x*_ZnFe_2_O_4_ + (8 − *x*)Li^+^ + (8 − *x*)e^−^ → Zn + 2Fe + 4Li_2_O

and

3 Li^+^ + Zn + e^−^ → LiZn.

The dealloying reaction of LiZn

4 LiZn → Li^+^ + Zn + e^−^

and oxidation reaction of Zn and Fe metallic states

5 Zn + Li_2_O → ZnO + 2Li^+^ + 2e^−^,

6 2Fe + 3Li_2_O → Fe_2_O_3_ + 6Li^+^ + 6e^−^,

occur during the charging process.^30^ These results are consistent with the characteristics of the CV curves. Fig. 6c displays the possible electrochemical mechanism of the ZnO–ZnFe_2_O_4_ composite electrode during the 1st discharge and charge cycle.

The lithiation and delithiation performances of ZnFe-3 at current densities of 1 and 5 A g^−1^ are shown in Fig. 6b. The electrodes exhibit excellent cycling performances and deliver capacities of 584 and 430 mA h g^−1^, respectively, after 200 discharge and charge cycles. After the initial cycle, the coulombic efficiency of the ZnFe-3 electrode remains nearly 100%. When the load current density was increased from 100 to 200, to 500, to 1000, and to 2000 mA g^−1^, the specific discharge capacities of ZnFe-3 were 945, 851, 804, 761, and 654 mA h g^−1^, respectively. When the current density returned to 100 mA g^−1^, the specific capacity could recover to 908 mA h g^−1^ (Fig. S3†). The capability of charge transfer and electrolyte diffusion at the electrode–electrolyte interface is evaluated by EIS. Fig. 6d presents a comparative study of the Nyquist plots for the ZnO–ZnFe_2_O_4_ composites in the fresh half-cell. All the curves show a semicircle in the high-frequency region corresponding to charge transfer impendence (*R*_ct_) and a straight line in the medium-frequency region for Warburg impendence (*R*_w_), and in the low-frequency region there also exists the solution resistance (*R*_s_). The *R*_s_ and *R*_ct_ values of the composites are significantly improved compared to bare ZnFe-1. ZnFe-3 exhibits lower *R*_s_ and *R*_ct_ in these electrodes, which is consistent with the previous electrochemical results. At the same time, the diffusion coefficient of ZnFe-3 is the largest among all the samples (Fig. S4†). The high specific capacity and rate capability of ZnO–ZnFe_2_O_4_ micro-cubic composites may be attributed to the following factors. Firstly, the hetero-structure electrode, composed of materials with different band-gap energies, *i.e.* n-type ZnO and p-type ZnFe_2_O_4_, will enhance the internal electric field at the hetero-interfaces, resulting in an improvement in the surface reaction kinetics and facilitating charge transport to improve the electrochemical activity. Secondly, the combination of dealloying/alloying and conversion reactions in an integrated hetero-structure electrode material will enhance the specific capacity. Thirdly, the hetero-junction between ZnO and ZnFe_2_O_4_ ensures high electrical conductivity and superior chemical–thermal stability, thereby improving its rate capability. Lastly, the synthesis strategy comprising crystal-seed induction followed by hydrothermal reactions can optimize the morphology and good dispersion of the bi-functional components on the hetero-interface. The unique composition and hetero-interface structure of the composite improves its electrochemical performances to a great extent.

## Conclusions

In summary, a facile marriage of crystal-seed induction and hydrothermal reactions was performed to fabricate ZnO–ZnFe_2_O_4_ micro-cubic composites, which were designed as anode materials for LIBs. Benefiting from its unique structure and bi-functional components, the ZnO–ZnFe_2_O_4_ micro-cubic composite exhibited outstanding electrochemical performance as an electrode material for LIBs. The as-prepared ZnO–ZnFe_2_O_4_ material can retain a specific capacity of 811 mA h g^−1^ after 200 cycles at a current density of 100 mA g^−1^. Even at 5 A g^−1^, the specific capacity can be stabilized at 430 mA h g^−1^ after 200 cycles. The synthesis strategy of crystal-seed induction to construct different transition metal oxides with distinct band-gap energies into hetero-structures of controllable morphology is favourable for adoption in rational design approaches for obtaining synergetic functionalities of electrode materials for LIBs.

## Conflicts of interest

There are no conflicts to declare.

## Supplementary Material

RA-008-C8RA01785A-s001
