# Supplementary material for: Crystal-seeds induced construction of ZnO–ZnFe2O4 micro-cubic composites as excellent anode materials for lithium ion battery
Source: RSC Adv. 2018 May 1;8(29):16187–92. doi: 10.1039/c8ra01785a (PMC9080250; doi:10.1039/c8ra01785a)
Supplement: RA-008-C8RA01785A-s001 [file RA-008-C8RA01785A-s001.pdf]

Electronic Supplementary Material (ESI) for RSC Advances.
This journal is © The Royal Society of Chemistry 2018

Electronic supplementary information (ESI)

Crystal-seeds induced construction of ZnO-$ZnFe_2O_4$ micro-cubic composites as excellent anode materials for lithium ion battery

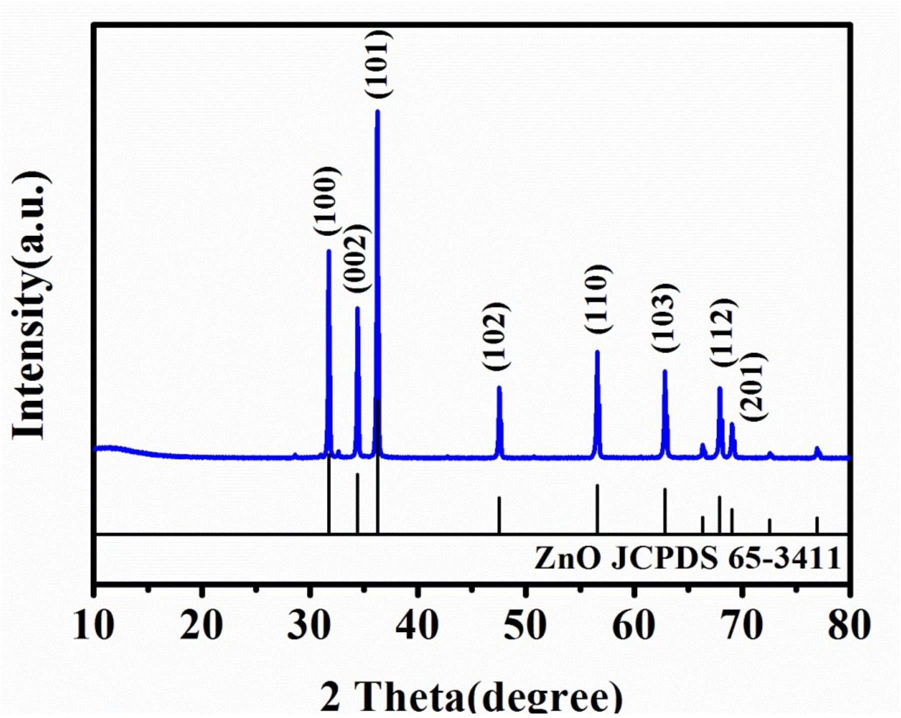

Fig. S1 XRD pattern of the ZnO seed

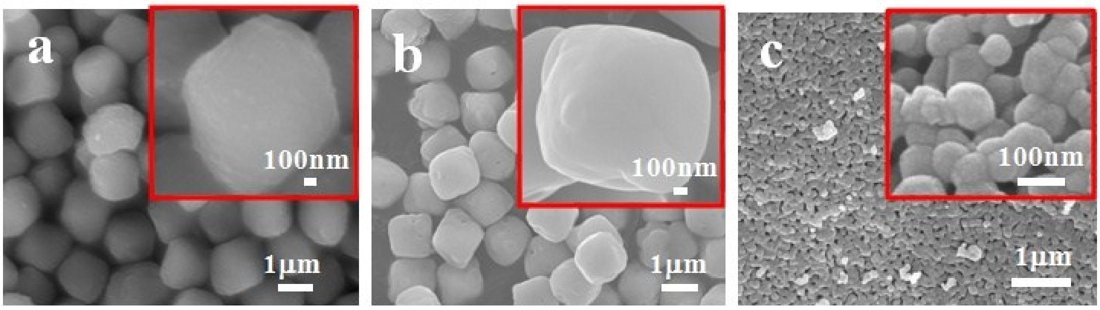

Fig. S2 SEM images of the ZnO seed(a), ZnFe-3-raw(b) and ZnFe-1(c)

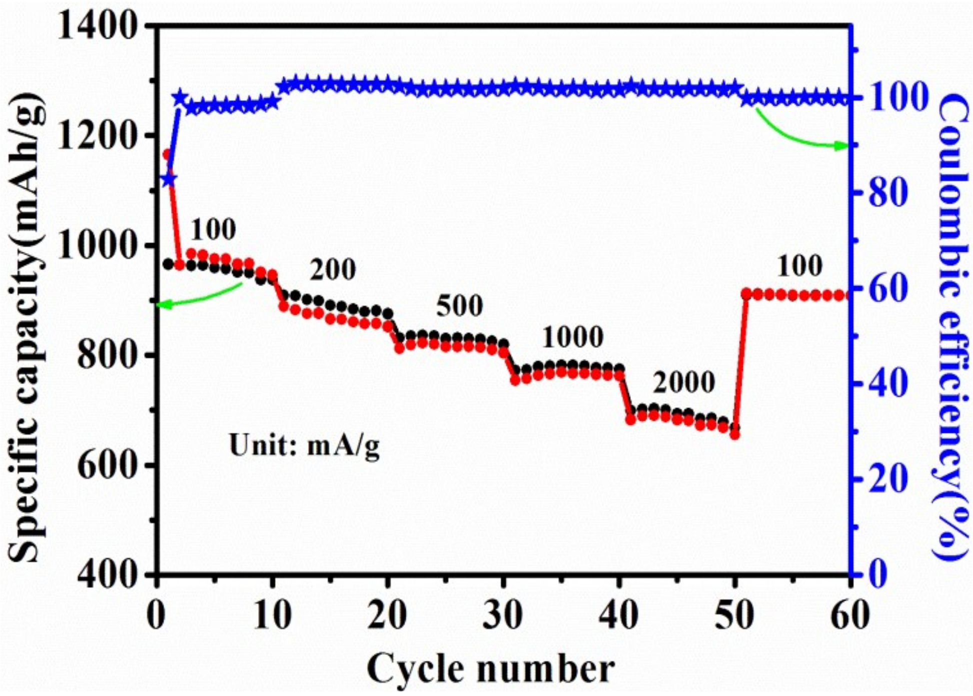

Fig. S3 The rate performance ZnFe-3 electrode

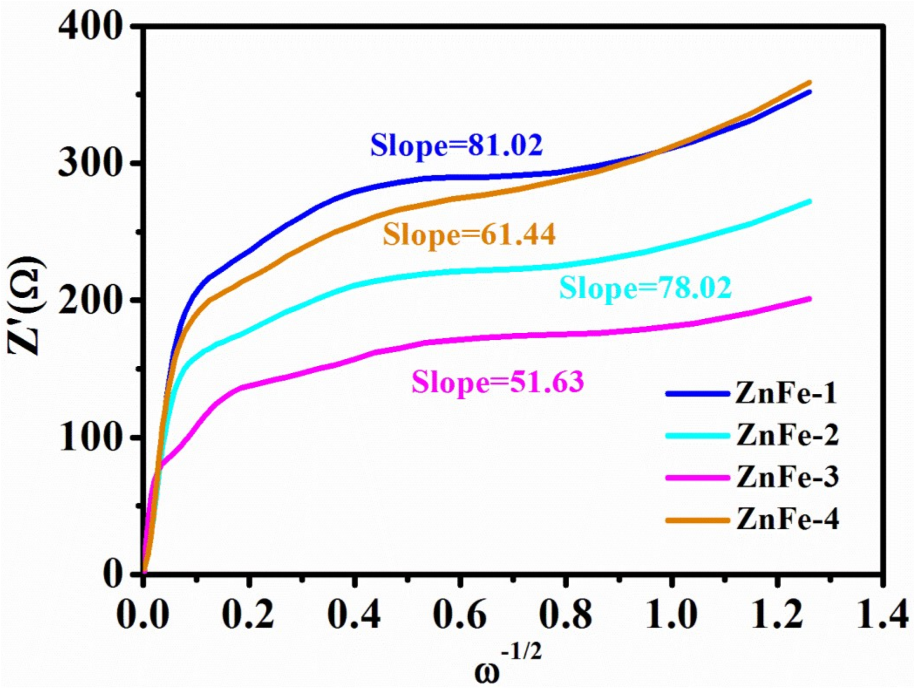

Fig. S4 The linear fitting of Warburg impedance of the ZnO-$ZnFe_2O_4$

Table S1 Electrochemical performances of ZnO or $ZnFe_2O_4$ materials previously reported for lithium-ion batteries

Materials	Current density ($A\cdot g^{-1}$)	Cycle number	Capacity ($mAh\cdot g^{-1}$)	Reference
$ZnFe_2O_4$ nanoparticles	0.116	50	over 800	Electrochim. Acta, 2011, 56: 9433–9438.
$ZnFe_2O_4$ Nano-Octahedrons	0.06	80	910	Nano. Res., 2012, 5: 477-485.
$ZnFe_2O_4$/C microspheres	0.05	100	1100	J. Power Sources 2014, 258: 305-313
nitrogen-doped carbon coated $ZnFe_2O_4$ composite	0.1	100	1477	Electrochim. Acta, 2015, 180: 622–628
$ZnFe_2O_4$ nanorods	0.1	50	983	J. Power Sources, 2016, 306: 718-723
ZnO/$ZnFe_2O_4$ porous nanoparticles	1	500	804	Chem. Eng. J., 2017, 308: 340–346
ZnO-$ZnFe_2O_4$ hollow microspheres	0.1	300	625	Electrochim. Acta, 2017, 249: 79–88
$ZnFe_2O_4$ nanoparticles /rGO composite	0.2	400	1106.7	J. Alloy. Compd., 2018, 737: 58-66
carbon-coated $ZnFe_2O_4$ nanoflakes	0.5	1000	778.6	Dalton Trans., 2018, 47: 3521-3529
**ZnO-$ZnFe_2O_4$ micro-cubic particles**	**0.1**	**200**	**811**	**In this work**
